# The chloroplast genome of an Endangered orchid species, *Gastrochilus calceolaris* (Orchidaceae: Aeridinae)

**DOI:** 10.1080/23802359.2018.1507646

**Published:** 2018-10-03

**Authors:** Fengxia Tang, Yu Song, Qiang Liu

**Affiliations:** a Center for Integrative Conservation, Xishuangbanna Tropical Botanical Garden, Chinese Academy of Sciences, Yunnan, China;; b College of Life Sciences, University of Chinese Academy of Sciences, Beijing, China;; c Lab of Ecology and Evolutionary Biology, State Key Laboratory for Conservation and Utilization of Bio-resource in Yunnan, Yunnan University, Yunnan, China

**Keywords:** *Gastrochilus calceolaris*, Endangered species, chloroplast genome, phylogenetic analysis

## Abstract

This study reported the complete chloroplast genome of a critically Endangered Orchidaceae species *Gastrochilus calceolaris* and its phylogenetic position in subtribe Aeridinae based on 12 orchid species plastomes. The plastome of *G. calceolaris*, with a 148,428 bp size, consisted of a pair of inverted repeat regions of 25,950 bp, a small single copy region of 11,139 bp, and a large single copy region of 85,389 bp. G + C content was 36.8%. The phylogenetic analysis highly supported the sisterhood between *Gastrochilus* and *Pelatantheria* and a monophyletic *Gastrochilus* group comprising of *G. calceolaris*, *G. fuscopunctatus*, and *G. japonicus.*

*Gastrochilus calceolaris* as the type species of genus of *Gastrochilus* (58 species included) is widely and variously distributed in the tropics and subtropics, mainly distributed in the southwest China, Bhutan, India, Malaysia, Indonesia, Nepal, Thailand and Vietnam at elevations of 1000–2700 m (Pathak et al. 2011). Currently, it has been listed as critically Endangered due to its geographical restricted distribution and the excessive collection (IUCN 2017). With rapid development of the second-generation sequencing technology, the chloroplast genome information was widely used for studying taxonomy, phylogeny, evolution, and ecology in plants (Jheng et al. 2012). However, few genomic data reported about *G. calceolaris* even the genus of *Gastrochilus*. Here, we displayed the first complete chloroplast genome of *G. calceolaris* deposited in GenBank (Accession Number: MH719016).

We used 5 g young leaves of *G. calceolaris* collected in the north of Myanmar to extract DNA accord to the modified CTAB method (Doyle and Dickson 1987). The voucher was deposited at the Biodiversity Research Group of Xishuangbanna Tropical Botanical Garden (Accession Number: XTBG-BRG-TFX0063). We used NGSQC Toolkitv2.3.3 software to filter the low-quality reads (Patel and Jain 2012). High-quality reads were de novo assembled into long reads (Contigs) by CLC Genomics Workbench 11.0 software (https://www.qiagenbioinformatics.com). The genome of *G. calceolaris* was assembled by using BioEdit software (http://www.mbio.ncsu.edu/bioedit/bioedit.html) accord to known reference chloroplast genomes *G. fuscopunctatus* and *G. japonicus* (Accession Number: KX871233 and KX871236). Then we used the Dual Organellar Genome Annotator (DOGMA) software to annotate the gene type of *G. calceolaris* (Wyman et al. 2004).

The chloroplast genome of *G.calceolaris*, with a 148,428 bp size, containing a pair of inverted repeats (IRs) of 25,950 bp, a small single copy (SSC) region of 11,139 bp and a large single copy (LSC) region of 85,389 bp. G+C content was 36.8%. In LSC, IR and SSC region, G+C content were 34.0, 43.1 and 28.2%, respectively. Additionally, we annotated 102 different genes, including 68 protein-coding genes, 30 tRNA genes and 4 rRNA genes. The NADH dehydrogenase (*ndh*) gene *ndhA*, *ndhB*, *ndhC*, *ndhD*, *ndhE* *ndhF*, *ndhG*, *ndhH*, *ndhI*, *ndhJ* and *ndhK* were pseudogenes. We detected 16 genes contained introns, amount which, 13 genes (*trnK-UUU*, *rps16*, *trnG-GCC*, *atpF*, *rpoC1*, *trnL-UAA*, *trnV-UAC*, *petB*, *petD*, *rpl16*, *rpl2*, *trnI-GAU*, and *trnA-UGC*) had a single intron, 3 genes (*ycf3*, *clpP*, and *rps12*) had two introns. *rpl22* gene straddled LSC/IRa border, and *ycf1* gene straddled IRa/SSC and SSC/IRb border.

To confirm the phylogenetic position of *G. calceolaris* in Aeridinae, We utilized 12 species chloroplast genomes of Aeridinae to construct the phylogenetic tree by using MEGA version 5.0 program (Tamura et al. 2011), *Thrixspermum japonicum* as outgroup. The plastomes were aligned by MAFFT version 7 software (Katoh and Standley 2013). A maximum likelihood method for phylogenetic analysis was performed base on GTR + I+G model in the RAxML version 8 program with 1000 bootstrap replicates (Darriba et al. 2012; Stamatakis 2014). In the phylogenetic tree (Figure 1), *G. Celceolaris* clustered with *G. fuscopunctatus* and *G. japonicus* as a monophyly with a 100% bootstrap value. The phylogenetic analysis highly supported the sisterhood of *Gastrochilus* and *Pelatantheria*.

**Figure 1.**
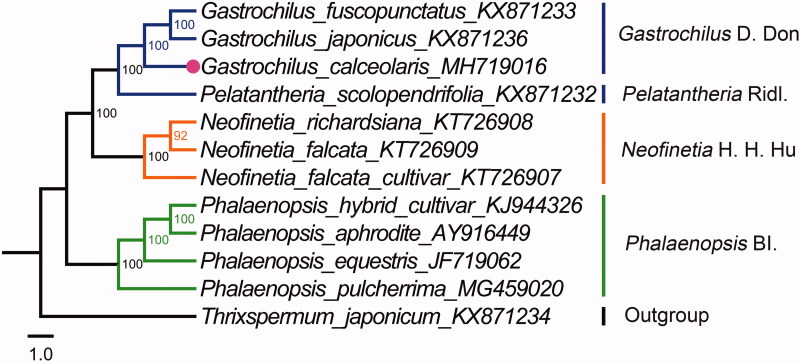
The ML phylogenetic tree for *G. calceolaris* based on 12 species chloroplast genomes in Aeridinae.

## Data Availability

The plastome data of the *G. calceolaris* will be submitted to Genebank of NCBI through the revision process. The accession numbers from Genebank must be supplied before the final acceptance of the manuscript.
